# Limitations in Activities of Daily Living in Community-Dwelling People Aged 75 and Over: A Systematic Literature Review of Risk and Protective Factors

**DOI:** 10.1371/journal.pone.0165127

**Published:** 2016-10-19

**Authors:** Anne van der Vorst, G. A. Rixt Zijlstra, Nico De Witte, Daan Duppen, Andreas E. Stuck, Gertrudis I. J. M. Kempen, Jos M. G. A. Schols, on behalf of the D-SCOPE Consortium

**Affiliations:** 1 Department of Health Services Research, CAPHRI School for Public Health and Primary Care, Maastricht University, Maastricht, the Netherlands; 2 Faculty of Psychology and Educational Sciences, Vrije Universiteit Brussel, Brussels, Belgium; 3 Faculty of Education, Health and Social Work, University College Ghent, Ghent, Belgium; 4 Department of Geriatrics, University Hospital, University of Bern, Bern, Switzerland; 5 Department of Family Medicine, CAPHRI School for Public Health and Primary Care, Maastricht University, Maastricht, the Netherlands; Nathan S Kline Institute, UNITED STATES

## Abstract

**Background:**

Most older people wish to age in place, for which functional status or being able to perform activities of daily living (ADLs) is an important precondition. However, along with the substantial growth of the (oldest) old, the number of people who develop limitations in ADLs or have functional decline dramatically increases in this part of the population. Therefore, it is important to gain insight into factors that can contribute to developing intervention strategies at older ages. As a first step, this systematic review was conducted to identify risk and protective factors as predictors for developing limitations in ADLs in community-dwelling people aged 75 and over.

**Methods:**

Four electronic databases (CINAHL (EBSCO), EMBASE, PsycINFO and PubMed) were searched systematically for potentially relevant studies published between January 1998 and March 2016.

**Results:**

After a careful selection process, 6,910 studies were identified and 25 were included. By far most factors were examined in one study only, and most were considered risk factors. Several factors do not seem to be able to predict the development of limitations in ADLs in people aged 75 years and over, and for some factors ambiguous associations were found. The following risk factors were found in at least two studies: higher age, female gender, diabetes, hypertension, and stroke. A high level of physical activity and being married were protective in multiple studies. Notwithstanding the fact that research in people aged 65 years and over is more extensive, risk and protective factors seem to differ between the ‘younger’ and ‘older’ olds.

**Conclusion:**

Only a few risk and protective factors in community-dwelling people aged 75 years and over have been analysed in multiple studies. However, the identified factors could serve both detection and prevention purposes, and implications for future research are given as well.

## Introduction

In 2013, 18% of the European Union (EU) population was aged 65 years or older, which is expected to increase to 28% in 2060. However, those aged 80 years and over are the fastest growing group with an expected increase from 5% in 2013 to 12% in 2060 [1]. With this rising proportion of the (oldest) old, concepts as active ageing and ageing in place have gained more and more attention [2]. According to the WHO [3], active ageing “aims to extend healthy life expectancy and quality of life for all people as they age”. Herewith, one of the key goals is maintaining independence, defined as, “the ability to perform functions related to daily living—i.e. the capacity of living independently in the community with no and/or little help from others” [3]. Indeed, delaying dependency is important to be able to live autonomously for as much and as long as possible [4], or in other words, to age in place. Ageing in place is not only important from a policy perspective (i.e. to reduce the high costs of institutionalisation; [5]), but is also the wish of most older people, even when significant health problems arise and they need care [6].

Early detection of risks associated with ageing is important to minimise or slow down negative consequences of ageing (e.g. [7]), and therewith to facilitate ageing in place. Researchers have been focusing on the process leading from ill health to disability for many years. A prominent model is the disablement process model, in which the progressive worsening from pathology (biochemical or physiological abnormalities) to impairments (dysfunctions affecting physical, mental, and/or social functioning), functional limitations (restrictions in performing activities), and eventually disability (difficulty doing activities) is elaborated. Furthermore, this model describes personal capacities and demands created by the social and physical environment that speed up or slow down this process, which can be both intra-individual (i.e. coping) and extra-individual (i.e. medical care) [8].

In a previous review with functional status decline as outcome measure, different risk factors related to the disablement process have been identified [9]. Amongst others, impaired cognition, depression, comorbidity, low frequency of social contacts, low level of physical activity, poor self-perceived health, smoking, and vision impairment have been found to influence this process [9]. An update and broadening of this review might give new insights for several reasons. First, it is important to examine a more specific, homogenous outcome to be able to determine a causal pathway. The results of the earlier review of Stuck et al. [9] were based on a broad definition of functional status decline, encompassing ADLs, instrumental ADLs (IADLs), and upper and lower extremity function. In contrast, the purpose of the present review was to specifically analyse the predictors of decline in ADLs, which are essential for an independent life [10]. This makes it possible to give a more focused contribution to preventive actions. Second, since the development of limitations in ADLs are known to increase with age, and especially in those aged 80 years and over (e.g. [11]), it seems more constructive to focus on the (oldest) old, while Stuck et al. [9] included studies with a broader age range (25+). For example, the need for help with ADLs was 3.5% in 65–74 year old Americans, 7.4% in those aged 75–84 years, and 18.1% in those aged 85 and over [12]. Since limitations in ADLs are most substantial in the oldest old, it is important to investigate whether it is possible to intervene in the slightly younger age group (75+) to work prevention-driven. Third, the identification of both risk and protective factors is relevant. As a result, strategies not only to reduce the risk factors can be implemented, but also to strengthen protective factors [13]. Although risk and protective factors are often thought to be different sides of the same coin, previous research has revealed differences between predictors for ill-health and excellent health [14]. In addition, Kempen et al. [15] showed that risk factors for and protective factors against IADLs and ADLs are not always that closely related. However, Stuck et al. [9] mainly focused on risk factors. Lastly, since the previous review dates from 1999, an update indeed is useful.

To conclude, this systematic review aims to obtain insight into risk factors for and protective factors against developing limitations in ADLs in community-dwelling people aged 75 and over.

## Materials and Methods

This systematic review was conducted according to the PRISMA (Preferred Reporting Items for Systematic Reviews and Meta-Analyses) guidelines [16]. The study protocol was not preregistered.

### Database sources and search strategy

Electronic databases CINAHL (EBSCO), EMBASE, PsycINFO and PubMed were searched on 7 March 2016 for manuscripts published between 1 January 1998 and 1 March 2016. Searches were tailored to the specific databases (S1 Text), and included key words and MeSH terms related to risk factors for and/or protective factors against developing limitations in ADLs in community-dwelling people aged 75 and over.

### Definition key concepts

ADLs were defined as activities essential for an independent life [10] or necessary for survival [8], representing common everyday tasks required for self-care [17]. Outcome measures needed to include at least three of the following activities: bathing, dressing, eating, toileting, and transferring (e.g. [17]).

Risk factors were defined as factors that lead to limitations in ADLs, whereas protective factors are associated with prevention or alleviation. To be as comprehensive as possible, all factors measured as potential risk factors for and/or protective factors against developing limitations in ADLs in community-dwelling older people were taken into account.

### Inclusion and exclusion criteria

Longitudinal, prospective studies, published in the public domain, written in English, and assessing risk factors for and/or protective factors against developing limitations in ADLs in community-dwelling people aged 75 and over were included. Studies in which all or part of the population was living in a long-term care facility at baseline were excluded as well, unless results for persons living at home at baseline were reported separately. Level of limitation in ADLs at baseline was no inclusion or exclusion criterion. Cross-sectional and intervention studies were excluded, as well as studies assessing subsamples of the population (e.g. people with sarcopenia), and case-control studies.

Studies evaluating IADLs, which are necessary for maintaining a dwelling in a given sociocultural setting (i.e. the ability to use the telephone; [8]), mobility, balance, gait performance, or lower and/or upper extremity function were excluded. Studies using combined measures of IADL and ADL were excluded as well, unless results were reported separately. In addition, studies in which people were defined as having developed limitations in ADLs when they were institutionalised, hospitalised or died at follow-up, were excluded.

### Selection and data extraction

Bibliographic details of retrieved studies were stored in EndNote (version X6) and the selection process was tracked in Excel. After removing duplicate records, a random sample of 10% was assessed by two reviewers (authors AvdV and DD). Agreement for inclusion and exclusion was greater than 95% (95.6%), and therefore, the process was completed by one reviewer (author AvdV). To decide upon inclusion, a predefined order was used to screen titles, abstracts, and keywords in the first stage, and full-texts if necessary, namely: population, concept and measurement of ADLs, design, measurement of risk and/or protective factors, and report of quantitative data. When one of the domains was scored as ‘exclude’, the study was excluded without assessing the other domains. When all domains were scored as ‘include’ and/or ‘unclear’, the full-text was screened using the same order. In case of doubt, the final decision was made after discussion with a third reviewer (author GARZ). Reference lists of included studies were reviewed to ensure inclusion of all relevant studies.

Data of a random selection of included studies (20%) were extracted by two independent reviewers (authors AvdV and DD). Because agreement was equal to the prearranged level of 95%, the data extraction was completed by one reviewer (author AvdV). A structured form with the following variables was used: (1) publication details (first author, year, country); (2) study details (baseline sample size, sample size in analyses, age, gender, length of follow-up, mortality rate); (3) concept and measurement of ADLs; (4) concept and measurement of risk and/or protective factors; and (5) quantitative results (If results were available for the entire sample, they were taken into account instead of subgroup analyses).

### Evaluation of studies and factors

#### Data synthesis and analyses

To structure this review, a direct content analysis approach was used ([18], for an overview). All risk and protective factors from a random sample of the included studies (n = 10) were identified and grouped into major domains as initial coding categories. Next, the risk and protective factors in all included studies were identified and grouped under one of these domains. If necessary, a domain was added. Meta-analyses were not performed because the identified factors were too heterogeneous.

#### Quality assessment and strength of evidence

Since there is limited consensus on the quality assessment of prognostic studies, a modified version of checklists by [19–20] was used (S1 Table). Selection, attrition, and measurement bias, confounding, and the risk of bias related to analyses and selective reporting of results were assessed. Two reviewers (authors AvdV and DD) independently assessed the quality of each study, and discussed disagreements. When not all the required information per domain was available, the item was scored as unclear.

The overall strength of evidence per factor (after grouping results) was determined using criteria based on the checklist used by Stuck et al. [9]. Studies with a quality assessment score of more than 50% (a score of ≥7 out of 12), and excluding people with limitations in ADLs at baseline were classified as high quality studies. Studies with a quality assessment score of 50% or less and/or including people with limitations in ADLs at baseline were classified as low quality studies. Ratings for overall evidence per factor were as follows:

- Evidence in ≥3 high quality studies: +++
- Evidence in 2 high or ≥3 low quality studies: ++
- Evidence in 1 high or 2 low quality studies: +
- Evidence in 1 low quality study: +/-

## Results

### Search outcome

**Fig 1:**
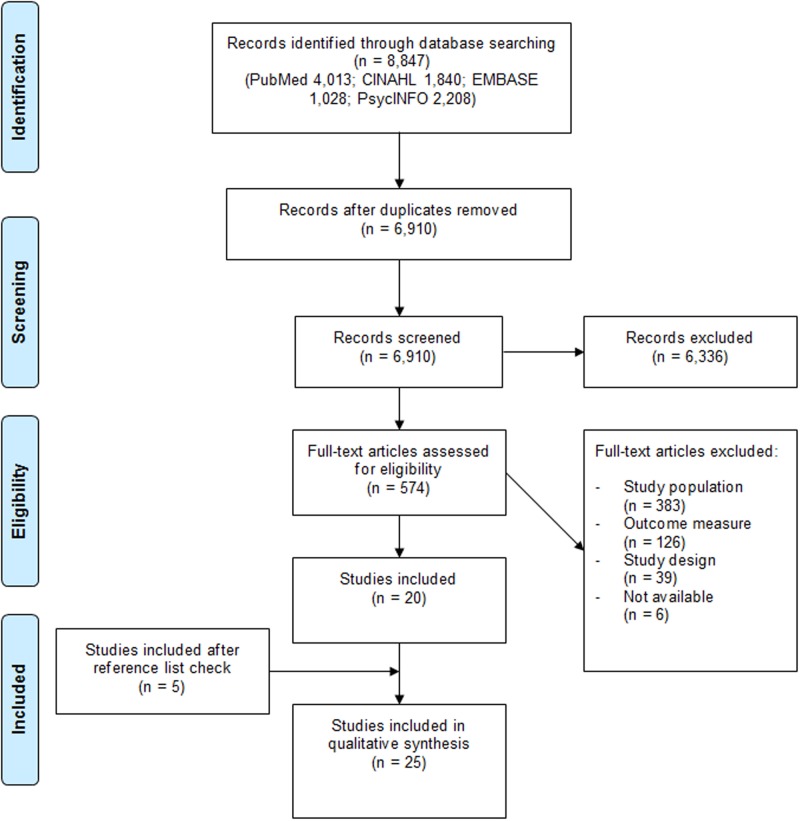
PRISMA flowchart. Figure credited from: Moher D, Liberati A, Tetzlaff J, Altman DG, The PRISMA Group (2009). Preferred Reporting Items for Systematic Reviews and Meta-Analyses: The PRISMA Statement. PLoS Med 6(7): e1000097. doi:10.1371/journal.pmed1000097

Fig 1 shows the flowchart of the selection process. Once duplicates were removed, 6,910 potentially relevant studies were identified. After assessing title, abstract, and keywords, 574 full-texts needed further examination, which resulted in the inclusion of 20 studies. After screening their reference lists, another five were included, resulting in 25 included studies.

### Description of included studies

Characteristics of the included studies are presented in Table 1. Studies were conducted in a wide range of countries. The length of follow-up ranged from 2 to 20 years. Limitations in ADLs were measured with questionnaires only (no performance-based assessments), and included the items bathing and dressing in all studies. Gender distribution was variable, whereby two studies included women only.

**Table 1:** Characteristics of included longitudinal studies predicting the development of limitations in ADLs over time*.

First author (year) Country	Main predictor examined	Years to prediction of limitations in ADLs (*n* intermediate follow-ups)	Baseline sample size (n), sample size(s) in analyses (%)	Mortality rate during follow-up (%)	Baseline mean age (SD)	Women (%)	ADLs included in measure	Exclusion of limitations at baseline	Quality assessment score (0–12)
Avlund (2002a) Denmark, Finland, Sweden [21]	Household composition	5	1,203 (61.8%)	25.6%	n/a, 75-year olds	59.4%	b,d,g,n,u		10
Avlund (2002b) Denmark, Finland [22]	Tiredness	5	835 (61.9%)	31.1%	n/a, 75-year olds	nr	b,d,n,u	yes	11
Avlund (2004) Denmark, Finland [23]	Social relations	5	651 (65.3%)	21.5%	n/a, 75-year olds	60.7%	b,d,n,u	yes	10
Black (2002) USA [24]	Cognition	2	601 (60.7%)	18.6%	nr, >75	62.7%	b,d,e,g,t,u,w	yes	10
Corona (2013) Brazil [25]	Weight	3	227 (70.0%)	8.4%	80.9 (nr)	100%	b,d,e,t,u,w	yes	7
Donald (1999) UK [26]	Falls	2 (1)	1,797 (70.8%, 55.3%)	25.3%	nr, >75	nr	b,d,e,u,w		10
Freedman (2008) USA [27]	Early-, mid-, and late-life factors	9 (3)	23,229 (79.9%)	nr	nr, >75	61.9	b,d,e,t,u,w		6
Fukutomi (2013) Japan [28]	Comprehensive geriatric assessment	2	527 (72.5%)	0.2%	81.1 (4.8)	60.3%	b,d,g,e,s,u,w		8
Gu (2004) China [29]	Socio-demographic characteristics	4 (1)	8,959 (53.0%); 11,161 (53.0%)	30.5%, 37.4%	nr, >80	nr	b,d,e,t,u		8
Guilley (2008) Switzerland [30]	Frailty	1.5	339 (77.9%)	49.3%	81.8 (nr)	nr	b,d,e,t,w		8
Houston (2011) USA [31]	Vitamin levels	3 (5)	1,677 (58.9%)	85.6%	85.2 (3.6)	64.5%	b,d,e,t,u	yes	7
Idland (2013) Norway [32]	Physical performance	9	300 (37.7%)	41.0%	79.5 (nr)	100%	b,d,e,t,u	yes	11
Jiang (2002) China [33]	Demographics	8 (2)	3,275 (83.0%, 61.5%, 48.5%)	46.6% (urban); 88.2% (hilly)	n/a, >75	51.1%	b,d,f,g,w,t		6
Landi (2007) Czech Republic, Denmark, Finland, France, Germany, Iceland, the Netherlands, Norway, Sweden, UK [34]	Physical activity	1	2,467 (81.3%)	nr	n/a, >80	78%	b,d,e,t,u	yes	9
Landi (2009) Italy [35]	Pain	2	248 (82.3%)	12.1%	84.3 (3.8)	66.1%	b,d,e,t,u	yes	7
Landi (2010) Italy [36]	Anorexia of aging, physical function	2	248 (82.7%)	12.1%	85.8 (4.8)	67.0%	b,d,e,t,w,u	yes	8
Li (2009) China [37]	Living arrangement	2	9,039 (53.1%)	37.0%	92.1 (7.74)	60%	b,c,d,e,t,u		7
Moody-Ayers (2005) USA [38]	Cognition, black-white differences	2	5,972 (95.0%)	9.3%	n/a, >80	63.6%	b,d,e,t,u		8
Okumiya (1999) Japan [39]	Gait/balance, manual dexterity	3	328 (90.5%)	nr	79.0 (nr)	63.6%	b,d,e,g,m,s,u,w	yes	8
Rantanen (2002) Denmark, Finland, Sweden [40]	Strength	5	821 (69.1%)	22.2%	n/a, 75-year olds	56.4%	b,d,t,u,w	yes	9
Sabayan (2012) the Netherlands [41]	Blood pressure	5 (4)	572 (nr)	nr	n/a, 85-year olds	66.8%	b,d,e,n,s,t,w,u		10
Shah (2012) USA [42]	Physical activity	5	718 (81.3%)	1.8%	n/a, >80	73.2%	b,d,e,t,u,w	yes	7
Stessman (2009) Israel [43]	Physical activity	7	1,861 (64.1%)	24.8%	n/a, >78	51.7%	b,c,d,e,t,u		9
Stessman (2014) Israel [44]	Loneliness	7	1,566 (93.9%)	21.8%	n/a, >78	46.6%	b,c,d,e,t,u		8
Sun (2009) China [45]	Rural and urban differences	4	8,635 (28.8%)	53.9%	88.7 (nr)	57.4%	b,c,d,e,t,u		8

* Sample size in analyses: multiple % when multiple analyses performed.

nr = not reported; n/a = not applicable. b = bathing/showering/washing/personal hygiene; c = continence/controlling bladder/bavel movement; d = dressing; e = eating/feeding; g = grooming; m = taking medicine; n = cutting/taking care of nails; s = climbing stairs; t = transferring (from bed to chair/in and out of bed/getting op from a chair); u = using the toilet; w = walking (across a room/around home)/locomotion.

Four studies met at least five of the six quality criteria, while for eight studies two criteria could not be judged because of a lack of information. In most cases, study participation and attrition could not be judged because it was not reported whether non-response was selective. Thirteen studies were classified as high quality (quality assessment score ≥7 out of 12, and excluding people with limitations in ADLs at baseline) (S2 Table for full details).

### Identified factors

Table 2 shows the overall findings grouped by domain (S3 Table for corresponding quantitative data). Factors that were associated with developing limitations in ADLs (irrespective of the quality score), or for which findings were ambiguous are described below. When evidence was found in at least two studies, it is mentioned explicitly.

**Table 2:** Synthesis of risk and protective factors for developing limitations in ADLs with combined strength of evidence per factor.

Domain	Specific factor(s)* (reference category if not the direct opposite)	Number of studies** (n risk, protective, indefinite, NSA)	Interpretation***	Combined strength of evidence****
**Socio-demographic characteristics**				
**Age**	Higher baseline age	7 (5,0,2,0)	Risk	+++
**Ethnicity / race**	African Americans / Hispanic Americans (Non-Hispanic whites) Non-Hispanic other / black / Hispanic (Hispanic white) Minor ethnicity Black (white)	4 (0,0,4,0)	Protective (subgroups)	+
**Gender**	Female gender	5 (4,0,1,0)	Risk	++
**Household composition**	Living alone	5 (0,0,2,3)	Unclear	
	Living with spouse / children / spouse and children / others	1 (0,0,1,0)	Risk	+/-
**Living environment**	Glostrup / Yyväskylä (Göteburg) Glostrup (Yyväskylä) Urban (rural) (Sub)urban (hilly) Midwest / Northeast / West (South, USA) Rural (urban)	6 (0,1,2,3)	Protective (rural) Risk (urban, Midwest; subgroups)	+/-
**Marital status**	Being married	4 (0,2,1,1)	Protective	+
	Being divorced/separated/widowed, and never married	1 (0,0,0,1)	NSA	+/-
**Place of birth**	Midwest / South / West / outside US (Northwest)	1 (0,0,1,0)	NSA	+/-
**Socio-economic characteristics**				
**Education**	Own education: ≤ 11 years ≤ 8 years 9–11 years (>high school) Education Years of education	4 (1,0,2,1)	Risk (not fully-adjusted)	++
	<8 years of mother’s education	1 (0,0,1,0)	Risk (not fully-adjusted)	+/-
**Housing tenure**	Not owning a house	1 (0,0,0,1)	NSA	+
**Income/wealth**	Lower income/wealth levels	1 (1,0,0,0)	Risk	+/-
	Having financial resources	2 (0,0,1,1)	Unclear	
**Occupation**	Operators, craftsmen, farmers / clerical and service industry workers / never worked / no lifetime occupation (white-collar) Being a veteran Non-agriculture / housewife (agriculture)	2 (0,0,1,0)	Risk	+
**Socioeconomic status as a child**	Poor/varied SES as child (well off/about average)	1 (0,0,1,0)	Risk (not fully-adjusted)	+/-
**Psychosocial factors**				
**Loneliness**	Feeling lonely	1 (0,0,0,1)	NSA	+/-
**Social participation**	Diversity in social relations Membership in club for retired people Paying and receiving visits, participating outside the home Not helping others, i.e. taking care of others, sewing, and make repairs Having weekly telephone contact with children Not going out at least once a week	3 (0,0,1,2)	NSA Risk (sewing)	+
**Receiving formal support**	Receiving support	1 (0,0,0,1)	NSA	+/-
**Receiving informal support**	Receiving support	1 (0,0,0,1)	NSA	+
**Self-reported conditions**				
**Anxiety**		1 (0,0,0,1)	NSA	+
**Arthritis**		2 (1,0,0,1)	NSA	+
**Cancer**		1 (1,0,0,0)	Risk	+/-
**Depression**		1 (0,0,0,1)	NSA	+
**Diabetes**		2 (2,0,0,0)	Risk	+
**Eye disorder**		1 (0,0,1,0)	Unadjusted risk	+
**Fractures**		2 (0,0,0,2)	NSA	++
**Heart disease**		3 (1,0,0,2)	NSA	++
**Hypertension**		3 (2,0,0,1)	Unclear	
**Lung disease**		2 (1,0,0,1)	NSA	+
**Number of chronic diseases**	0 versus 1 versus 2–8 0–13	2 (0,0,0,2)	NSA	++
**Psychiatric disorder**		1 (1,0,0,0)	Risk	+/-
**Stroke**		2 (2,0,0,0)	Risk	+
**Health behaviour**				
**Alcohol consumption**	Currently drinking alcohol	1 (0,0,0,1)	NSA	+/-
**Physical activity**	Performing activities weekly ≥ 2 / <2 hours (no activity) Hours a week4 hours / vigorous sports ≥ twice weekly (<4 hours) Involvement in activities Involved in physical exercise program	5 (0,4,1,0)	Protective	+++
**Smoking**	Not smoking	1 (0,1,0,0)	Protective	+/-
	Quitted smoking (never)	1 (0,0,0,1)	NSA	+/-
	Smoking (never)	1 (1,0,0,0)	Risk	+/-
**Observed health-related measures**				
**Cognition**	Digit span and -symbol, fluency, visual reproduction, Raven’s progressive matrices Errors on modified SPMSQ (Modified) MMSE	4 (1,0,0,3)	NSA	++
**Depression**	CES-D GDS Depression risk	3 (0,0,1,2)	NSA	++
**Blood pressure**	No hypertension	1 (1,0,0,0)	Risk	+
	Higher diastolic blood pressure (DBP)	1 (0,0,0,1)	NSA	+/-
	Higher mean arterial pressure (MAP)	1 (0,1,0,0)	Protective	+/-
	Higher pulse pressure (PP)	1 (0,1,0,0)	Protective	+/-
	Higher systolic blood pressure (SBP)	1 (0,1,0,0)	Protective	+/-
**Frailty**	≥ 2 affected frailty domains	1 (1,0,0,0)	Risk	+/-
**In need of long-term care**	Based on physical strength, nutritional status, oral function, houseboundness, cognition, and depression risk	1 (0,0,0,1)	NSA	+/-
**Limitations in IADLs**	≥ 1 out of 7 activities	1 (0,0,1,0)	Unadjusted risk	+
**Limitation in gait/balance**		1 (1,0,0,0)	Risk	+
**Limited muscle strength**	Low grip strength	2 (1,0,0,1)	Unclear	
	Low elbow flexion strength	1 (1,0,0,0)	Risk	+
	Low knee extension strength	1 (1,0,0,0)	Risk	+
	Low trunk extension strength	1 (0,0,0,1)	NSA	+
	Low trunk flexion strength	1 (1,0,0,0)	Risk	+
**Number of chronic diseases**	2–7 (0–1)	2 (1,0,1,0)	Unadjusted risk	+
**Other physical function limitation**				
	Poor functional reach	1 (0,0,1,0)	Risk (not fully-adjusted)	+
	Poor physical strength	1 (0,0,0,1)	NSA	+/-
	Low manual dexterity	1 (1,0,0,0)	Risk	+
	Step climbing	1 (0,0,1,0)	Risk (not fully-adjusted)	+
	Tiredness in activities	1 (0,0,1,0)	Risk	+
	Low walking speed	1 (1,0,0,0)	Risk	+
**Weight**				
	Weight gain	1 (1,0,0,0)	Risk	+
	Weight loss	1 (0,0,1,0)	Unadjusted risk	+
**Vitamin status**				
	25(OH)D vitamin status: ng/ML <20.0 / 20.0–29.9	1 (0,0,0,1)	NSA	+/-
**Self-reported health-related measure**				
**Falls**		1 (0,0,0,1)	NSA	+/-
**Cognition**	Question-based (e.g. “do others point you to forgetfulness?)	1 (0,0,0,1)	NSA	+/-
**Hearing**	Good / fair/poor hearing (excellent/very good)	1 (0,0,1,0)	Risk	+/-
**Medication**	High medication use	1 (0,0,0,1)	NSA	+
**Pain**	Experiencing daily / multiple site / moderate to severe pain (no pain)	1 (0,0,1,0)	Risk	+
**Peak stature**	Based on self-reported height	1 (0,0,1,0)	Risk (not fully-adjusted)	+/-
**Self-rated health**	Fair/poor / good / very good (excellent)	4 (0,0,2,2)	Unclear	
**Self-rated health as a child (recalled)**	Fair/poor / good / very good (excellent)	1 (0,0,1,0)	Risk	+/-
**Subjective well-being**	E.g. quality of life and happiness	1 (0,0,0,1)	NSA	+/-
**Vision**	Impaired vision Vision less than excellent/very good	2 (1,0,1,0)	Risk	+/-
**Weight / nutrition**	Obesity (BMI ≥ 30)	1 (1,0,0,0)	Risk	+/-
	Weight loss Unintentional BMI Anorexia of ageing	3 (0,0,2,1)	Unclear	
**Other**				
**Proxy response**		1 (1,0,0,0)	Risk	+/-

* Underlined: statistical significant risk or protective factor (in case multiple categories were examined / instruments were used)

** Risk = statistical significant increased risk for developing limitations in ADLs; Protective = statistical significant decreased risk for developing limitations in ADLs; Indefinite = findings differ per model, and/or per subgroup (e.g. Gu & Yi [29] reported findings per age group and by gender); NSA = no statistically significant association

*** Unclear = different findings across studies; NSA = highest combined strength of evidence for studies that did not found statistically significant associations

**** +++ = evidence in ≥ 3 high quality studies; ++ = evidence in 2 high / ≥3 low quality studies; + = evidence in 1 high / 2 low quality studies; +/- = evidence in 1 low quality study

#### Socio-demographic characteristics

*Higher age* was a risk factor in multiple studies, whereby it should be noted that two studies taking age into account used data from the Chinese Longitudinal Healthy Longevity Survey (CLHLS) [29, 45]. *Being from a minor ethnicity/race* was protective in women aged 90 years and over, and men in their eighties (e.g. [29]), though once only after controlling for cognition [38]. *Female gender* was a risk in multiple studies. Findings regarding *household composition* were ambiguous. Studies using data from the Nordic Research on Ageing (NORA) study found that *living alone* was not statistically significant associated with developing limitations in ADLs [22–23], while *sustained living alone* was a risk factor [21]. *Living with children* and *with those other than children and spouse* were risk factors [37]. *Living in a rural area* was protective [45], and *living in an urban area* was a risk factor [29] (both used data from the CLHLS study). *Living in the South of the USA* was a risk factor [27]. Out of two studies that were based on the CLHLS, one performed subgroup analyses, and found that *being married* was a risk in women aged 90–99, but protective in men aged 90–99 (only unadjusted) [29]. In two other studies, being married was protective.

#### Socio-economic characteristics

*Fewer years of education* was a risk factor in multiple studies, although not in most adjusted models (e.g. [24, 27]). While two studies used data from the CLHLS study, education was an unadjusted risk factor only when performing subgroup analyses in those aged 80–89 [29]. *Fewer years of mother’s education* became nonsignificant after controlling for late-life factors (e.g. marital status, income, and chronic conditions) [27]. People with *lower income* levels (<19,999 dollars) and *low total wealth* (<49,999 dollars) were at risk for developing limitations in ADLs [27], whereas *economic independence* was protective in women in their nineties [29]. Females who were *housewives* had an increased risk for developing limitations in ADLs, as well as people *without a lifetime occupation* [29]. *Poor socioeconomic status as a child* was no longer a risk factor after correcting for late-life factors [27].

#### Psychosocial factors

Regarding *social participation*, not sewing for others was a risk factor in women [23].

#### Health behaviour

*High levels of physical activity* were protective in multiple studies (e.g. [24]). *Smoking* was a risk factor, while *not smoking* was protective [27, 45].

#### Self-reported conditions

Findings regarding *arthritis* were ambiguous. *Cancer* was a risk factor [27]. *Diabetes* was a risk in multiple studies [24, 27]. Having *eye disorders* was an unadjusted risk [32]. Self-reported *hypertension* was a risk in two out of three studies [24, 27]. Findings regarding *lung disease* were ambiguous. Having a *psychiatric disorder* was a risk factor [27], as well as *stroke* in two studies [24, 27].

#### Observed health-related measures

*Cognition* and *depression* were examined multiple times, but were statistically significant associated with developing limitations in ADLs only once, namely when examined with the Short Portable Mental Status Questionnaire (SPMSQ) [24], respectively the Geriatric Depression Scale (GDS) [25]. *No hypertension* was a risk factor [39], while other *blood pressure measures* (e.g. high mean arterial pressure) were protective [41]. People with *multiple impaired frailty domains* (e.g. mobility capacities, and memory problems) were at risk for developing limitations in ADLs [30]. *IADL disability* was an unadjusted risk factor [25]. *Impaired gait/balance* [39] and *low elbow flexion*, *knee extension*, *and trunk flexion strength* were risk factors [40]. Findings regarding grip strength were ambiguous. Two studies examining *chronic conditions* used data from the NORA study took different confounding factors into account, and found different results [21–22]. *Poor functional reach* lost its significant association after controlling for step climbing and walking speed [32]. *Low manual dexterity* was a risk factor [39]. *Step climbing* lost its significant association after controlling for functional reach and walking speed [32]. *Tiredness in activities* was a risk factor in all models but one; the statistically significant association disappeared after controlling for health factors [22]. *Low walking speed* [32] and *weight gain* were risk factors. *Weight loss* was an unadjusted risk [25].

#### Self-reported health-related measure

*Poor hearing* was a risk factor [27]. *Moderate to severe pain* was a risk, as well as *daily* and *multiple site pain*, although the last two were only significant in unadjusted models [35]. *Peak stature* was no longer associated after controlling for mid- and late-life factors [27]. Findings regarding *self-rated health* were ambiguous. *Less than excellent self-rated health as a child* (recalled) was a risk factor, as well as *vision less than excellent/very good* [27]. *Obesity* [27] was a risk factor, whereas for weight loss ‘*anorexia of ageing*’ was an unadjusted risk factor [36].

#### Other

*Response by a proxy* was a risk factor [27].

## Discussion

This systematic review aimed to identify risk factors for and protective factors against developing limitations in ADLs in community-dwelling people aged 75 years and over. Higher age, female gender, diabetes, hypertension, and stroke were risk factors in at least two studies. In addition, quite a number of risk factors were supported by only one study (e.g. frailty). However, some risk factors were no longer associated in (fully) adjusted models (e.g. IADL disability). With respect to overall domains, lower socio-economic status in old age seems to predict limitations in ADLs, more than socio-economic status at younger ages (e.g. [27]). In addition, people with more self-reported clinical conditions, and more health-related problems, both observed (e.g. limited strength) and self-reported (e.g. pain), seem at risk for developing limitations in ADLs, although most sub-factors have been examined only once.

A high level of physical activity and being married were found to be protective. In addition, some factors were protective in one study only (e.g. economic independence). When considering socio-demographic characteristics as a whole, being from a minor ethnicity/race seems to be protective.

Although numerous factors have been examined, most do not seem to be able to predict the development of limitations in ADLs (e.g. never married, anxiety, and falls).

### Comparison with previous review

When comparing the current findings with the outcomes of the review by Stuck et al. [9], it should be noted that different data was used (i.e. studies published prior to 1999 versus studies published since then). Furthermore, the previous review had a broader age range, as well as a broader outcome measure. Therefore, a substantial higher number of studies (78) were included in their review. In the review from 1999, substantial evidence (in at least two high quality studies) was found for associations between risk factors and functional decline that have not been found in the current review, but were examined in the studies that were part of the present review (e.g. depression, alcohol consumption, and cognition). Additionally, Stuck et al. [9] found substantial empirical evidence for risk factors for which we found evidence in one study only (e.g. high BMI and smoking), and for factors for which we found ambiguous results (e.g. poor self-rated health). These differences might be explained by the difference in age range and outcome measure.

### The ‘oldest old’ versus those aged 65 years and over

Out of 25 included studies, six performed subgroup analyses across different age groups [33, 34, 38, 42, 43, 44]. Some found differences, while others did not. For example, Moody-Ayers et al. [38] found that differences in developing limitations in ADLs between black and white people primarily disappeared in those aged 80 years and over compared to those aged 70–79, but did emerge after controlling for cognition. On the other hand, Landi et al. [34], who examined the effect of physical activity on limitations in ADLs, found no differences between those aged 65–79 and those 80 years and over. Overall, there seem to be some differences between age groups. Therewith, different prevention strategies might be needed for different age groups.

Since numerous studies in those aged 65 years and over emerged while including and excluding articles for the current review, it seemed reasonable to compare some of the studies in those aged 65 and over (including people aged 75 and over) with the findings of this review. It appears that there are factors that have been examined in those aged 65 years and over, but not in those aged 75 years and over. For example, performing volunteer or paid work (e.g. [46]) and positive affect (e.g. [47]) were found to be protective, and not being satisfied with your social network was a risk factor in those aged 65 years and over (e.g. [48]).

### Ambiguous findings

Some findings were different for different measurement methods. For example, cognition was no risk factor when measured with the Mini-Mental State Examination (MMSE) and as self-report, but was when examined with the SPMSQ [24]. However, it is known that additional neuropsychological testing besides the MMSE is needed for diagnostics [49–50], and that self-reported cognition correlates poorly with actual neuropsychological performance (e.g. [51]). However, the use of different covariates might explain some of the contradictory results as well. For example, Gu and Yi [29] controlled for other and more variables than Black and Rush [24] while examining being married.

For some findings it might be important to examine possible underlying factors. For example, living with children and with those other than spouse or children was a risk factor [37], but sustained living alone was a risk factor as well [21]. One might argue that living alone may result in the maintenance of functioning because people are forced to perform all activities by themselves. However, previous research has shown that women who lost their spouse did not feel obliged to perform activities anymore and, consequently, became less active ([52], in [21]), while it is known that being physically active is important to prevent the development of limitations in ADLs (e.g. [36]).

### Limitations and strengths

This review has several limitations. First, it was difficult to determine the actual quality of each study since not all the necessary information was reported. Second, low ratings of strength of evidence must be interpreted with some caution because this was the result of a lack of data for some factors. In addition, inappropriate sample sizes might have influenced the results. For example, for weight loss, no statistically significant association was found, though the 95%CI ranged from 0.8 to 4.62 [25]. This may indicate underpowered, low quality studies. Lastly, since five studies could be included only after a reference check, it cannot be ruled out with certainty that all relevant studies have been included.

This review has several strengths as well. First, cross-sectional and retrospective studies were excluded. Factors that precede the development of limitations in ADLs could, therefore, be distinguished from factors that result from these limitations; and the natural course could be determined. Second, by examining a specific outcome (limitations in ADLs) in a specific population (people aged 75 years and over), the explanatory potential of the identified factors may be considered as higher. Although it can be argued that findings are not generalizable to the entire older population, this specific focus and knowledge is needed for prevention and intervention strategies that are more tailored to this specific vulnerable population. Lastly, by using strict criteria for strength of evidence, it was made certain that revealed factors were supported by multiple studies, which enlarged the strength of evidence.

## Conclusion

Five risk (higher age, female gender, diabetes, hypertension, and stroke), and two protective factors (being married and being physical active) were empirically supported by at least two studies. However, most factors were examined in one study only, while some were associated only in unadjusted analyses, and for other factors ambiguous results or no statistically significant associations were found. Factors that may help to identify groups at risk (e.g. older women, people living with children) have been identified, as well as risk (e.g. obesity) and protective factors (e.g. physical activity) for which preventive actions can take place.

### Implications for future research

More specific research in community-dwelling people aged 75 and over is needed (1) to investigate risk and protective factors that have not yet been examined in multiple studies but do appear to be related with developing limitations in ADLs; and (2) to examine factors that have been found to influence the development of limitations in ADLs in those aged 65 years and over. Factors that are useful to detect vulnerable groups of older people (e.g. related to potential risk factors such as income, and household composition), as well as those older people for which preventive actions can take place (e.g. the possible risk factor psychiatric disorder) should be further investigated. In addition, future research (1) could perform subgroup analyses to compare different age groups since we noticed that subgroup analyses did reveal differences between the ‘younger’ and ‘older’ olds; (2) could focus more on protective factors because this is a neglected area, and it is shown that they are not always the other side of the coin (e.g. being married was protective, but never married was not significantly associated); and (3) could examine the interaction between risk factors.

### Implications for clinical practice

Several risk factors that have been identified could serve for the detection of groups at risk for developing limitations in ADLs. Thereafter, preventive actions need to take place. When it is possible to intervene on the risk factors itself, for example by trying to prevent the occurrence of adverse clinical conditions, or to cure them, this is preferable. However, some of the revealed risk factors cannot be influenced and therefore could serve detection purposes only (older age, and female gender). In that case, it is important to take preventive actions that are known to decrease the risk of developing limitations in ADLs. Being physically active is, to our knowledge, the best studied intervention, and found to be effective, whereby it can slow-down the process of disability as well ([53] for an overview). Such intervention is in line with our approach of not merely focusing on risk factors, but also looking at protective factors, which can be promoted, as the most prominent protective factor that appeared in this review was physical activity. Thus, promoting an active lifestyle in general is important. Less demanding physical activities, such as household chores, walking, and gardening might be attractive even for older people with (less severe) limitations. The WHO (2012) further notes the importance of having a motivating environment to support older people to actually play an active role. In this perspective, age-friendly cities might be important to make sure places are suitable for older people to perform leisure activities [54].

## Supporting Information

S1 PRISMA Checklist (DOC)

S1 Text Search strategy per database. (DOCX)

S1 Table Quality assessment form. (DOCX)

S2 Table Quality assessment per study. (DOCX)

S3 Table Data synthesis—statistical findings per study and overall interpretation per domain. (DOCX)
