# Correction: Limitations in Activities of Daily Living in Community-Dwelling People Aged 75 and Over: A Systematic Literature Review of Risk and Protective Factors

**DOI:** 10.1371/journal.pone.0170849

**Published:** 2017-01-23

**Authors:** Anne van der Vorst, G. A. Rixt Zijlstra, Nico De Witte, Daan Duppen, Andreas E. Stuck, Gertrudis I. J. M. Kempen, Jos M. G. A. Schols, on behalf of the D-SCOPE Consortium

The third author’s name is incorrect in the citation. The correct citation is: van der Vorst A, Zijlstra GAR, De Witte N, Duppen D, Stuck AE, Kempen GIJM, et al. (2016) Limitations in Activities of Daily Living in Community-Dwelling People Aged 75 and Over: A Systematic Literature Review of Risk and Protective Factors. PLoS ONE 11(10): e0165127. doi:10.1371/journal.pone.0165127
