# Supplementary material for: Limitations in Activities of Daily Living in Community-Dwelling People Aged 75 and Over: A Systematic Literature Review of Risk and Protective Factors
Source: PLoS One. 2016 Oct 19;11(10):e0165127. doi: 10.1371/journal.pone.0165127 (PMC5070862; doi:10.1371/journal.pone.0165127)
Supplement: S1 Text — (DOCX) [file pone.0165127.s002.docx]

**S1 Text.** Search strategy per database

**CINAHL (EBSCO)**

((MH "Risk Assessment") OR (MH "Prognosis") OR (protective factors) OR (protecting) OR (protective) OR (protects) OR (risk factors) OR (risk) OR (factor) OR (predictor) OR (predictors) OR (predicts) OR (predicting) OR (association) OR (associations) OR (associated) OR (correlation) OR (correlations) OR (correlated) OR (correlates) OR (relation) OR (related) OR (relationship) OR (relates) OR (causal) OR (causality) OR (change) OR (changes) OR (changing) OR (life-course)) AND ((MH "Disabled") OR (MH "Activities of Daily Living") OR (MH "Instrumental Activities of Daily Living (Saba CCC)") OR (MH "Instrumental Activities of Daily Living Alteration (Saba CCC)") OR (MH "Activities of Daily Living Alteration (Saba CCC)") OR (MH "Activities of Daily Living (Saba CCC)") OR (MH “Functional Status”) OR (disabled persons) OR (disability) OR (daily living activity) OR (instrumental activities of daily living) OR (instrumental activity of daily living) OR (ADL) OR (IADL) OR (functional status) OR (functional abilities) OR ((impaired) OR (limited) OR (decline) AND (function)) ((MH "Community Living") OR (community dwelling) OR (home living) OR (aging in place)) NOT ((MH “Cross Sectional Studies” OR (MH "Experimental Studies"))

Limiters: language: Dutch/Flemish, English, German. Age: Aged: 65+ years, Aged, 80 and over. Human.

**EMBASE**

((protection/ or protecting.mp. or protective.mp. or protects.mp. or risk factors/ or risk/ or factor.mp. or predictor.mp. or predictors.mp. or predicts.mp. or predicting.mp. or association.mp. or associations.mp. or associated.mp. or correlation.mp. or correlations.mp. or correlated.mp. or correlates.mp. or relation.mp. or related.mp. or relationship.mp. or relates.mp. or causal.mp. or causality.mp. or change.mp. or changes.mp. or changing.mp. or life-course.mp.) and (disabled person/ or daily life activity/ or disability.mp. or disabled.mp. or daily living activity.mp. or instrumental activities of daily living.mp. or instrumental activity of daily living.mp. or ADL.mp. or IADL.mp. or functional status.mp. or functional abilities.mp. or ((impaired or limited or decline) and function).mp.) and (independent living/ or community dwelling.mp or home living.mp or community living.mp or aging in place.mp) and (cohort analysis/ or prospective study/ or observational study/ or follow up/ or retrospective study/)) not (cross-sectional study/ or intervention study/)

Limits: human, Dutch or English or German, and aged <65+ years

**PsycINFO**

((DE "Risk Factors") OR (DE "Protective Factors") OR (protective factors) OR (protecting) (“protective”) OR (protects) OR (risk factors) OR (risk) OR (factor) OR (predictor) OR (predictors) OR (predicts) OR (predicting) OR (association) OR (associations) OR (associated) OR (correlation) OR (correlations) OR (correlated) OR (correlates) OR (relation) OR (related) OR (relationship) OR (relates) OR (causal) OR (causality) OR (change) OR (changes) OR (changing) OR (life-course)) AND ((disabled) OR (disabled persons) OR (disability) OR (DE "Activities of Daily Living") OR (DE "Daily Activities") OR (instrumental activities of daily living) OR (DE "Ability Level") OR (daily living activity) OR (instrumental activities of daily living) OR (instrumental activity of daily living) OR (ADL) OR (IADL) OR (functional status) OR (functional abilities) OR ((impaired) OR (limited) OR (decline) AND (function))) AND ((community living) OR (community dwelling) OR (home living) OR (aging in place))

Limiters: language: Dutch, English German. Age: 65 years and older, 85 years and older. Population: human, (fe)male. Source types: academic journals

**PubMed**

((protective factors [Majr]) OR (protect* [Tiab]) OR (risk factors [Majr]) OR (risk[MeSH]) OR (risk* [Tiab]) OR (factor) OR (predictor) OR (predictors) OR (predicts) OR (predicting) OR (association) OR (associations) OR (associated) OR (correlation) OR (correlations) OR (correlated) OR (correlates) OR (relation) OR (related) OR (relationship) OR (relates) OR (causal) OR (causality) OR (change) OR (changes) OR (changing) OR (life-course)) AND ((disabled persons [MeSH:NoExp]) OR (activities of daily living[MeSH]) OR (disabled) OR (disability) OR (daily living activity) OR (instrumental activities of daily living) OR (instrumental activity of daily living) OR (ADL) OR (IADL) OR (functional status) OR (functional abilities) OR ((impaired) OR (limited) OR (decline) AND (function))) AND ((community dwelling) OR (home living) OR (community living) OR (aging in place)) AND ((cohort studies [MesH]) OR (prospective Studies[MesH) OR (observational study [Publication type]) OR (follow-up studies [MesH]) OR (retrospective studies[MesH]))

NOT ((cross-sectional studies [MesH]) OR (intervention studies[MesH]))

Filters activated: Humans, Dutch, English, German, Aged: 65+ years, 80 and over: 80+ years.
