# Supplementary material for: Limitations in Activities of Daily Living in Community-Dwelling People Aged 75 and Over: A Systematic Literature Review of Risk and Protective Factors
Source: PLoS One. 2016 Oct 19;11(10):e0165127. doi: 10.1371/journal.pone.0165127 (PMC5070862; doi:10.1371/journal.pone.0165127)
Supplement: S1 Table — (DOCX) [file pone.0165127.s003.docx]

**S1 Table.** Quality assessment form

| **Biases** | **Study methods (to facilitate the consensus process)** | **Rating** |
| --- | --- | --- |
| **Study participation** |  |  |
| **Goal**: to judge the risk of selection bias. The study sample must represent the population of interest regarding key characteristics (e.g. age, sex).   - Low risk: participation rate is at least 75% and non-response is not selective (data presented). - Medium risk: participation rate is at least 75% but non-response is selective, or participation rate is less than 75% but non-response is not selective - High risk: participation rate is <75% and non-response is selective - Unclear: insufficient information (study participation is not described / not described in sufficient detail to allow a definite judgement, i.e. participation rate and/or selectivity is unknown) |  |  |
| **Study attrition (not including mortality)** |  |  |
| Goal: to judge the risk of attrition bias. Baseline characteristics for the group lost to follow-up and the analyzed group must be similar, or studies must had a good attrition rate.   - Low risk: response rate at the main moment of follow-up is at least 80 percent and loss to follow-up is not associated with key characteristics (data presented). - Medium risk: response rate is at least 80% but loss-to-follow-up is selective, or attrition rate is lower than 80% but not selective. - High risk: response rate is <80% and is associated with key characteristics - Unclear risk: insufficient information (study attrition is not described / not described in sufficient detail to allow a definite judgement, i.e. participation rate and/or selectivity is unknown) |  |  |
| **Outcome measurement** |  |  |
| **Goal**: judge the risk of measurement bias.   - Low risk: the measurement method is standardized, valid and reliable (consistent and reproducible): citations or discussion by the authors why the use of the measurement is valid/reliable, e.g. use of available standards or evidence from similar studies - Medium risk: standardized, and validated scales, but different procedures per participants; or no records on validity etcetera, but method is described in sufficient detail and is therefore reproducible. - High risk: no records on validity and reliability, not reproducible. |  |  |
| **Prognostic factor measurement** |  |  |
| **Goal**: to judge the risk of measurement bias.   - Low risk: the measurement method is standardized, valid and reliable (consistent and reproducible): citations or discussion by the authors why the use of the measurement is valid/reliable, e.g. use of available standards or evidence from similar studies - Medium risk: standardized, and validated scales, but different procedures per participants; or no records on validity etcetera, but method is described in sufficient detail and is therefore reproducible. - High risk: no records on validity and reliability, not reproducible. |  |  |
| **Confounding** |  |  |
| **Goal**: to judge the risk of bias due to confounding.   - Low risk: confounders are accounted for / multivariable analyses were performed. - High risk: confounders are not accounted for / no multivariable analysis were performed - Unclear risk: insufficient information (not described / not described in sufficient detail to allow a definite judgement) |  |  |
| **Analysis** |  |  |
| **Goal**: to judge the risk of bias related to inappropriate analysis and invalid results.   - Low risk: OR’s or RR’s reported, no selective reporting of results - Medium: selective reporting of results, or no report of OR’s / RR’s - High risk: selective reporting of results |  |  |
