# Supplementary material for: Limitations in Activities of Daily Living in Community-Dwelling People Aged 75 and Over: A Systematic Literature Review of Risk and Protective Factors
Source: PLoS One. 2016 Oct 19;11(10):e0165127. doi: 10.1371/journal.pone.0165127 (PMC5070862; doi:10.1371/journal.pone.0165127)
Supplement: S2 Table — (DOCX) [file pone.0165127.s004.docx]

**S2 Table.** Quality assessment per study^*^

| **Author (year)** | **Study participation** | **Study attrition** | **Outcome measure** | **Prognostic** | **Confounding** | **Analyses** | **Total score** | **Total unclear** |
| --- | --- | --- | --- | --- | --- | --- | --- | --- |
| Avlund (2002a) | 2 | 0 | 2 | 2 | 2 | 2 | 10 | 0 |
| Avlund (2002b) | 2 | 1 | 2 | 2 | 2 | 2 | 11 | 0 |
| Avlund (2004) | 2 | 0 | 2 | 2 | 2 | 2 | 10 | 0 |
| Black (2002) | 2 | ? | 2 | 2 | 2 | 2 | 10 | 1 |
| Corona (2013) | ? | 1 | 1 | 1 | 2 | 2 | 7 | 1 |
| Donald (1999) | 2 | 1 | 2 | 2 | 2 | 1 | 10 | 0 |
| Freedman (2008) | ? | ? | 1 | 1 | 2 | 2 | 6 | 2 |
| Fukutomi (2013) | 1 | 1 | 2 | 1 | 2 | 1 | 8 | 0 |
| Gu (2011) | ? | ? | 2 | 2 | 2 | 2 | 8 | 2 |
| Guilley (2008) | ? | ? | 2 | 2 | 2 | 2 | 8 | 2 |
| Houston (2011) | ? | 0 | 1 | 2 | 2 | 2 | 7 | 1 |
| Idland (2013) | 1 | 2 | 2 | 2 | 2 | 2 | 11 | 0 |
| Jiang (2002) | ? | 1 | 2 | 1 | 1 | 1 | 6 | 1 |
| Landi (2007) | ? | 1 | 2 | 2 | 2 | 2 | 9 | 1 |
| Landi (2009) | ? | ? | 2 | 1 | 2 | 2 | 7 | 2 |
| Landi (2010) | 1 | ? | 2 | 2 | 2 | 1 | 8 | 1 |
| Li, 2009 | ? | ? | 1 | 2 | 2 | 2 | 7 | 2 |
| Moody-Ayers (2005) | ? | 1 | 1 | 2 | 2 | 2 | 8 | 1 |
| Okumiya (1999) | ? | ? | 2 | 2 | 2 | 2 | 8 | 2 |
| Rantanen (2002) | ? | 1 | 2 | 2 | 2 | 2 | 9 | 1 |
| Sabayan (2012) | 2 | ? | 2 | 2 | 2 | 2 | 10 | 1 |
| Shah (2012) | ? | ? | 2 | 2 | 2 | 1 | 7 | 2 |
| Stessman (2009) | ? | 1 | 2 | 2 | 2 | 2 | 9 | 1 |
| Stessman (2014) | ? | ? | 2 | 2 | 2 | 2 | 8 | 2 |
| Sun (2009) | ? | 1 | 1 | 2 | 2 | 2 | 8 | 1 |

^*^ ? = Not sufficient information, 0 = high risk, 1 = medium risk, 0 = low risk.
