# Supplementary material for: Limitations in Activities of Daily Living in Community-Dwelling People Aged 75 and Over: A Systematic Literature Review of Risk and Protective Factors
Source: PLoS One. 2016 Oct 19;11(10):e0165127. doi: 10.1371/journal.pone.0165127 (PMC5070862; doi:10.1371/journal.pone.0165127)
Supplement: S3 Table — (DOCX) [file pone.0165127.s005.docx]

**S3 Table**. Data synthesis – statistical findings per study and overall interpretation per domain *

|  | **First author (year)** | **Specific definition / measurement methods / categories** | **Statistical findings** | **Interpretation** |
| --- | --- | --- | --- | --- |
| **Socio-demographic characteristics** |  |  |  |  |
| **Age** |  |  |  |  |
|  | Black (2002) | Baseline age | OR model 2 = **1.04 (1.01-1.06)** | Risk |
|  | Corona (2013) | Baseline age | OR model 1 = **1.10 (1.02-1.18)**  OR model 2 = 1.05 (0.97-1.13) | Unadjusted risk |
|  | Freedman (2008) | 80-84 versus 75-79 | OR model 1 = **1.54**  OR model 2 = **1.53**  OR model 3 = **1.51**  OR model 4 = **1.47** | Risk |
|  |  | 85+ versus 75-79 | OR model 1 = **3.10**  OR model 2 = **3.11**  OR model 3 = **2.99**  OR model 4 = **2.89** | Risk |
|  | Gu (2004) | 80-89 | OR model 2 = **1.10**  OR model 3 = **1.08** (men)  OR model 2 = **1.08**  OR model 3 = **1.06** (women) | Risk |
|  |  | 90-99 | OR model 2 = **1.08**  OR model 3 = **1.06** (men)  OR model 2 = **1.08**  OR model 3 = **1.06** (women) | Risk |
|  |  | 100-105 | OR model 2 = **1.18**  OR model 3 = **1.18** (men)  OR model 2 = 1.07  OR model 3 = 1.03 (women) | Risk in men |
|  | Idland (2013) | Baseline age | OR model 1 **= 1.20 (1.04-1.39)**  OR model 2 **= 1.22 (1.04-1.44)**  OR model 3 **= 1.20 (1.02-1.41)**  OR model 4 **= 1.19 (1.01-1.41)**  OR model 5 **= 1.19 (1.01-1.41)** | Risk |
|  | Okumiya (1999) | Baseline age | OR model 1 **= 1.1 (1.0-1.1)** | Risk |
|  | Sun (2009) | Baseline age | β = .02, p **<.01** | Risk |
| **Ethnicity / race** |  |  |  |  |
|  | Black (2002) | African Americans compared to Non-Hispanic whites | OR model 2 = **0.69 (0.50-0.95)** | Protective |
|  |  | Hispanic Americans compared to Non-Hispanic whites | OR model 2 = 0.82 (0.53-1.26) | Not associated |
|  | Freedman (2008) | Non-Hispanic other versus non-Hispanic white | OR model 2 = 1.03  OR model 3 = 0.99  OR model 4 = 0.86 | Not associated |
|  |  | Non-Hispanic black versus non-Hispanic white | OR model 2 = **1.25**  OR model 3 = 1.18  OR model 4 = 0.97 | Risk, but no longer after controlling for mid- and late-life factors |
|  |  | Hispanic versus non-Hispanic white | OR model 2 = 1.11  OR model 3 = 0.90  OR model 4 = **0.77** | Protective, only after controlling for late-life factors |
|  | Gu (2004) | Minor ethnicity versus not, age 80-89 | OR model 2 = **0.57**  OR model 3 = **0.41** (men)  OR model 2 = 0.88  OR model 3 = 0.88 (women) | Protective in men |
|  |  | Minor ethnicity versus not, age 90-99 | OR model 2 = 0.71  OR model 3 = 0.69 (men)  OR model 2 = **0.69**  OR model 3 = **0.61** (women) | Protective in women |
|  |  | Minor ethnicity versus not, age 100-105 | OR model 2 = 0.80  OR model 3 = 0.68 (men)  OR model 2 = **0.38**  OR model 3 = **0.39** (women) | Protective in women |
|  | Moody-Ayers (2005) | Black versus white | OR model 1 = 1.08 (0.70-1.66)  OR model 2 = 1.15 (0.77-1.73)  OR model 3 = 1.08 (0.70-1.67)  OR model 4 = 0.96 (0.61-1.52)  OR model 5 = 0.90 (0.56-1.42)  OR model 6 = **0.61 (0.38-0.96)** | Protective, but only after controlling for cognition |
| **Gender** |  |  |  |  |
|  | Avlund (2002b) | Women versus men | OR model 1 **= 1.7 (1.0-2.7)**  OR model 2 = 1.5 (0.9-2.5) | Unadjusted risk |
|  | Black (2002) | Women versus men | OR model 2 = **1.32 (1.01-1.72)** | Risk |
|  | Freedman (2008) | Women versus men | OR model 1 = **1.58**  OR model 2 = **1.57**  OR model 3 = **1.45**  OR model 4 = **1.33** | Risk |
|  | Jiang (2002) | Women versus men | Time serie analyses: **p < .01** | Risk |
|  | Sun (2009) | Women versus men | β = .16, **p < .05** | Risk |
| **Household composition** |  |  |  |  |
| Living alone | Avlund (2002b) | Living alone versus not | OR model 1 = 0.8 (0.5-1.2)  OR model 6 = 0.8 (1.5-1.3) | Not associated |
|  | Avlund (2004) | Living alone versus not | OR model 1 = 1.0 (0.4-2.9) (men)  OR model 1 = 1.0 (0.6-1.8) (women) | Not associated |
|  | Idland (2013) | Living alone versus not | OR model 1 = **0.24 (0.07-0.88)**  OR model 2 = 0.34 (0.10-1.28)  OR model 3 = 0.40 (0.10-1.56)  OR model 4 = 0.30 (0.08-1.12) | Unadjusted risk |
|  | Sun (2009) | Living alone versus not | β = -.04, p >.05 | Not associated |
|  | Avlund (2002a) | Became alone between age 70-75 versus sustained living with others | OR model 1 = 4.0 (0.7-21.5)  OR model 2 = 4.4 (0.7-26.9) | Not associated |
|  | Avlund (2002a) | Sustained living alone versus sustained living with others | OR model 1 = **3.9 (1.4-10.7)**  OR model 2 = **4.0 (1.3-12.2)** | Risk |
| Living with others | Li (2009) | With spouse versus alone | OR model 2 = 1.15 (0.84-1.56) | Not associated |
|  |  | With children versus alone | OR model 2 = **1.33 (1.07-1.66)** | Risk |
|  |  | With spouse and children versus alone | OR model 2 = 1.25 (0.92-1.71) | Not associated |
|  |  | With others than spouse/children versus alone | OR model 2 = **2.12 (1.28-3.49)** | Risk |
| **Living environment** |  |  |  |  |
|  | Avlund (2002a) | Glostrup versus Göteburg | OR model 2 = 0.7 (0.2-2.4) | Not associated |
|  |  | Jyväskylä versus Göteburg | OR model 2 = 0.8 (0.2-3.3) | Not associated |
|  | Avlund (2002b) | Glostrup versus Jyväskylä | OR model 1 = 0.9 (0.6-1.4)  OR model 2 = 0.9 (0.6-1.5) | Not associated |
|  | Gu (2004) | Urban versus rural area, age 80-89 | OR model 2 = 1.011  OR model 3 = 0.941 (men)  OR model 2 = 0.950  OR model 3 = 1.043 (women) | Not associated |
|  |  | Urban versus rural area, age 90-99 | OR model 2 = 1.067  OR model 3 = 1.130 (men)  OR model 2 = **1.209**  OR model 3 = **1.284** (women) | Risk in women |
|  |  | Urban versus rural area, age 100-105 | OR model 2 = 0.912  OR model 3 = 0.933 (men)  OR model 2 = **1.293**  OR model 3 = **1.382** (women) | Risk in women |
|  | Jiang (2002) | Living in an (sub)urban or hilly region | Time serie analyses: all but one ns | Not associated |
|  | Freedman (2008) | Midwest versus South | OR model 4 = **1.18** | Risk |
|  |  | Northeast versus South | OR model 4 = 1.11 | Not associated |
|  |  | West versus South | OR model 4 = 1.11 | Not associated |
|  | Sun (2009) | Living in a rural area | β = -.26, **p <.001** | Protective |
| **Marital status** |  |  |  |  |
| Being married | Black (2002) | Being married versus not | OR model 2 = **0.46 (0.35-0.61)** | Protective |
|  | Gu (2004) | Being married versus not, age 80-89 | OR model 2 = 0.869  OR model 3 = 1.055 (men)  OR model 2 = 0.925  OR model 3 = 0.954 (women) | Not associated |
|  |  | Being married versus not, age 90-99 | OR model 2 = **0.766**  OR model 3 = 0.857 (men)  OR model 2 = **1.667**  OR model 3 = **1.838** (women) | Risk in women, protective in men in unadjusted analysis |
|  |  | Being married versus not, age 100-105 | OR model 2 = 1.006  OR model 3 = 1.026 (men)  OR model 2 = 1.087  OR model 3 = 1.192 (women) | Not associated |
|  | Jiang (2002) | Being married versus not | Time serie analyses: **p < .01** | Protective |
|  | Sun (2009) | Being married versus not | β = .01, p >.05 | Not associated |
| Being divorced/separated/ widowed, and never have been married | Freedman (2008) | Being divorced/separated/widowed versus being married | OR model 4 = 1.04 | Not associated |
|  |  | Never have been married versus being married | OR model 4 = 1.00 | Not associated |
| **Place of birth** |  |  |  |  |
|  | Freedman (2008) | Midwest versus Northwest | OR model 2 = 1.10  OR model 3 = 1.07  OR model 4 = 1.10 | Not associated |
|  |  | South versus Northwest | OR model 2 **=** **1.34**  OR model 3 **=** **1.25**  OR model 4 = 1.19 | Mixed |
|  |  | West versus Northwest | OR model 2 = 1.11  OR model 3 = 1.13  OR model 4 = 1.22 | Not associated |
|  |  | U.S. territory/outside the United States versus Northwest | OR model 2 = 0.89  OR model 3 = 0.84  OR model 4 = 0.90 | Not associated |
| **Socio-economic characteristics** |  |  |  |  |
| **Education** |  |  |  |  |
| Years of own education | Black (2002) | Less than 12 versus 12 years or more | OR model 2 = **2.45 (1.88-3.19)** | Risk |
|  | Freedman (2008) | 8 or fewer years versus more than high school | OR model 3 = **1.64**  OR model 4 = 1.07 | Risk, but not after controlling for late-life factors |
|  |  | 9-11 years versus more than high school | OR model 3 = **1.20**  OR model 4 = 0.83 | Risk, but not after controlling for late-life factors |
|  |  | High school versus more than high school | OR model 3 = 1.07  OR model 4 = 0.91 | Not associated |
|  | Gu (2004) | Education versus no education, aged 80-89 | OR model 2 = **0.80**  OR model 3 = 0.86 (men)  OR model 2 = **0.74**  OR model 3 = 0.84 (women) | Risk, but not in fully-adjusted model |
|  |  | Education versus no education, aged 90-99 | OR model 2 = 0.84  OR model 3 = 0.85 (men)  OR model 2 = 1.10  OR model 3 = 1.17 (women) | Not associated |
|  |  | Education versus no education, aged 100-105 | OR model 2 = 0.69  OR model 3 = 0.76 (men)  OR model 2 = 0.78  OR model 3 = 0.87 (women) | Not associated |
|  | Sun (2009) | Years of school completed | β = -.01, p > .05 | Not associated |
| Years of mother’s education | Freedman (2008) | Fewer than eight versus more than eight years | OR model 2 = **1.21**  OR model 3 = **1.11**  OR model 4 = 1.06 | Risk, but not after controlling for late-life factors |
|  |  | Missing versus more than eight years | OR model 2 = 1.16  OR model 3 = 1.03  OR model 4 = 1.04 | Not associated |
| **Housing tenure** |  |  |  |  |
|  | Avlund (2002b) | Renters versus owners | OR model 1 = 12.8 (0.9-5.6)  OR model 5 = 2.4 (0.8-7.4) | Not associated |
|  |  | Sheltered housing versus owners | OR model 1 = 1.5 (0.95-2.5)  OR model 5 = 1.4 (0.9-2.3) | Not associated |
| **Income / wealth** |  |  |  |  |
| Lower income / wealth levels | Freedman (2008) | Couple income ≤ v9,999 versus 50,000+ | OR model 4 = **1.39** | Risk |
|  |  | Couple income 10,000-19,999 versus 50,000+ | OR model 4 = **1.21** | Risk |
|  |  | Couple income 20,000-29,999 versus 50,000+ | OR model 4 = 1.13 | Not associated |
|  |  | Couple income 30,000-39,999 versus 50,000+ | OR model 4 = 0.97 | Not associated |
|  |  | Couple income 40,000-49,999 versus 50,000+ | OR model 4 = 0.91 | Not associated |
|  | Freedman (2008) | Total wealth ≤ 1,000 versus 300,000+ | OR model 4 = **1.85** | Risk |
|  |  | Total wealth 2,000-49,999 compared to 300,000+ | OR model 4 = **1.33** | Risk |
|  |  | Total wealth 50,000-149,999 versus 300,000+ | OR model 4 = 1.12 | Not associated |
|  |  | Total wealth 150,000-299,999 versus 300,000+ | OR model 4 = 1.12 | Not associated |
| Having financial resources | Gu (2004) | Economic independence versus dependence, age 80-89 | OR model 2 = **0.78**  OR model 3 = 0.97 (men)  OR model 2 = 0.99  OR model 3 = 1.01 (women) | Protective in men in unadjusted analysis only |
|  |  | Economic independence versus dependence, age 90-99 | OR model 2 = 0.89  OR model 3 = 0.98 (men)  OR model 2 = **0.55**  OR model 3 = **0.58** (women) | Protective in women |
|  |  | Economic independence versus dependence, age 100-105 | OR model 2 = 1.84  OR model 3 = 1.67 (men)  OR model 2 = 0.67  OR model 3 = 0.70 (women) | Not associated |
|  |  | Adequate sources to maintain daily costs versus inadequate, age 80-89 | OR model 2 = 0.92  OR model 3 = 0.99 (men)  OR model 2 = 1.06  OR model 3 = 1.22 (women) | Not associated |
|  |  | Adequate sources to maintain daily costs versus inadequate, age 90-99 | OR model 2 = 0.95  OR model 3 = 1.11 (men)  OR model 2 = 0.94  OR model 3 = 1.09 (women) | Not associated |
|  |  | Adequate sources to maintain daily costs versus inadequate, age 100-105 | OR model 2 = 1.21  OR model 3 = 1.48 (men)  OR model 2 = 1.24  OR model 3 = 1.20 (women) | Not associated |
|  | Sun (2009) | Receiving money from children versus not | β = .04, p > .05 | Not associated |
| **Occupation** |  |  |  |  |
|  | Freedman (2008) | Operators, craftsmen, and farmers versus white-collar (professionals, managers, salesmen) | OR model 3 = 1.05  OR model 4 = 0.96 | Not associated |
|  |  | Clerical and service industry workers versus white-collar | OR model 3 = 1.01  OR model 4 = 0.93 | Not associated |
|  |  | Never worked versus white-collar | OR model 3 = 1.15  OR model 4 = 1.00 | Not associated |
|  |  | No lifetime occupation (job held longer than 5 years) versus white-collar | OR model 3 = **1.36**  OR model 4 = **1.19** | Risk |
|  |  | Being a veteran versus not | OR model 3 = 1.00  OR model 4 = 1.01 | Not associated |
|  | Gu (2004) | Non-agriculture versus agriculture, age 80-89 | OR model 2 = 1.01  OR model 3 = 1.07 (men)  OR model 2 = 1.12  OR model 3 = 1.14 (women) | Not associated |
|  |  | Non-agriculture versus agriculture, age 90-99 | OR model 2 = 1.07  OR model 3 = 1.20 (men)  OR model 2 = 1.24  OR model 3 = 1.19 (women) | Not associated |
|  |  | Non-agriculture versus agriculture, age 100-105 | OR model 2 = 0.95  OR model 3 = 1.13 (men)  OR model 2 = 1.09  OR model 3 = 0.81 (women) | Not associated |
|  |  | Housewife versus agriculture, age 80-89 | OR model 2 = **1.41**  OR model 3 = **1.38** | Risk |
|  |  | Housewife versus agriculture, age 90-99 | OR model 2 = **1.25**  OR model 3 = **1.31** | Risk |
|  |  | Housewife versus agriculture, age 100-105 | OR model 2 = 1.088  OR model 3 = 1.095 | Not associated |
| **Socioeconomic status as a child** |  |  |  |  |
|  | Freedman (2008) | Poor/varied socioeconomic status versus well off/about average as a child (recalled) | OR model 2 = **1.19**  OR model 3 = **1.15**  OR model 4: 0.98 | Risk, but not after controlling for late-life factors |
| **Psychosocial factors** |  |  |  |  |
| Loneliness | Stessman (2014) | Not feeling lonely versus feeling lonely (rarely – very often) | OR model 1 = 1.12 (0.61-2.05)  OR model 2 = 0.71 (0.34-1.52)  (ADL dependence)  OR model 1 = 0.82 (0.44-1.54)  OR model 2 = 0.69 (0.34-1.40)  (ADL difficulty) | Not associated |
| Social participation | Avlund (2002b) | Number of categories with whom subjects have contact at least once per week (range 0-6) | OR model 1 = 1.5 (0.0.95-2.5)  OR model 6 = 1.4 (0.9-2.3) | Not associated |
|  | Avlund (2004) | Membership in a club for retired people (yes/no) | OR model 1 = 1.8 (0.7-4.3)  OR model 2 = 2.1 (0.8-5.7) (men) | Not associated |
|  |  | Paying visits to others, receiving visits at home, and participating in activities outside the home (range 0-3) | OR model 1 = 1.1 (0.9-1.6) (women) | Not associated |
|  |  | Number of categories with whom subjects have contact at least once per week (range 0-6) | Bivariate analyses: *ns* | Not associated |
|  |  | Not helping others (take care of, have on vacation, sew, make repairs, do housework) | OR model 1 = 0.5 (0.2-1.3) (men)  OR model 1 = **2.1 (1.1-4.3)**  OR model 2 = **2.7 (1.2-5.7)** (women) | Risk in women |
|  |  | Having weekly telephone contact with children versus less often | OR model 1 = 1.6 (0.9-3.0)  OR model 2 = 1.5 (0.8-3.0) (women) | Not associated |
|  | Fukutomi (2013) | Being housebound versus not | Not further examined | Not associated |
| Receiving formal support | Avlund (2002a) | Receiving help from local health service system versus not | OR model 2 = 1.4 (0.5-4.4) | Not associated |
| Receiving informal support | Avlund (2004) | Receiving social support (housework, cooking,  shopping, and repairs in the house by relatives or friends) versus not | Not further examined | Not associated |
| **Self-reported conditions** |  |  |  |  |
| **Anxiety** |  |  |  |  |
|  | Idland (2013) | Self-reported, diagnosed | p = .91, not examined in logistic regression models | Not associated |
| **Arthritis** |  |  |  |  |
|  | Freedman (2008) |  | OR model 4 = **2.38** | Risk |
|  | Idland (2013) | Self-reported, diagnosed | p = .09, not examined in logistic regression models | Not associated |
| **Cancer** |  |  |  |  |
|  | Freedman (2008) | Self-reported, diagnosed | OR model 4 = **1.17** | Risk |
| **Depression** |  |  |  |  |
|  | Idland (2013) | Self-reported, diagnosed | p = .73, not examined in logistic regression models | Not associated |
| **Diabetes** |  |  |  |  |
|  | Black (2002) | Self-reported, physician-diagnosed | OR model 2 = **1.69 (1.18-2.42)** | Risk |
|  | Freedman (2008) | Self-reported, diagnosed | OR model 4 = **1.33** | Risk |
| **Eye disorder** |  |  |  |  |
|  | Idland ( 2013) | Impaired vision versus not. Self-reported, **diagnosed** | OR model 1 = **3.33 (1.19-9.30)**  OR model 2 = 2.61 (0.82-8.16)  OR model 3 = 2.52 (0.85-7.50)  OR model 4 = 2.17 (0.70-6.75) | Risk in unadjusted analysis only |
| **Fractures** |  |  |  |  |
|  | Idland (2013) | Self-reported, diagnosed | p = .37, not examined in logistic regression models | Not associated |
|  | Black (2002) | Hip fracture, self-reported, physician-diagnosed | OR model 2 = 1.49 (0.22-1.95) | Not associated |
| **Heart disease** |  |  |  |  |
|  | Black (2002) | Self-reported, physician-diagnosed | OR model 2 = 1.24 (0.97-1.58) | Not associated |
|  | Freedman (2008) | Self-reported, diagnosed | OR model 4 = **1.36** | Risk |
|  | Idland (2013) | Self-reported, diagnosed | p = .06, not examined in logistic regression models | Not associated |
|  |  |  |  |  |
| **Hypertension** |  |  |  |  |
|  | Black (2002) | Self-reported, physician-diagnosed | OR model 2 = **1.85 (1.47-2.33)** | Risk |
|  | Freedman (2008) | Self-reported, diagnosed | OR model 4 = **1.15** | Risk |
|  | Idland (2013) | Self-reported, diagnosed | p = .80, not examined in logistic regression models | Not associated |
| **Lung disease** |  |  |  |  |
|  | Freedman (2008) | Self-reported, diagnosed | OR model 4 = **1.65** | Risk |
|  | Idland (2013) | Self-reported, diagnosed | p = .26, not examined in logistic regression models | Not associated |
| **Number of chronic diseases** |  |  |  |  |
|  | Corona (2013) | Self-reported (2-8 versus 1 versus 0) | Only descriptive baseline characteristics by 3-year weight change: p >0.05 | Not associated |
|  | Sun (2009) | Self-reported (0-13) | β = -.01, p > .05 | Not associated |
| **Psychiatric disorder** |  |  |  |  |
|  | Freedman (2008) | Self-reported, diagnosed | OR model 4 = **2.13** | Risk |
| **Stroke** |  |  |  |  |
|  | Black (2002) | Self-reported, physician-diagnosed | OR model 2 = **2.98 (2.15-4.13)** | Risk |
|  | Freedman (2008) |  | OR model 4 = **2.83** | Risk |
| **Health behaviour** |  |  |  |  |
| **Alcohol consumption** |  |  |  |  |
|  | Sun (2009) | Currently drinking alcoholic beverages yes/no | β = .-01, p > .05 | Not associated |
| **Physical activity** |  |  |  |  |
|  | Avlund (2002b) | Performing activities (walking, active sports, gardening) less often than weekly versus weekly | OR model 1 = **3.0 (1.6-5.6)**  OR model 6 **= 2.9 (1.5-5.5)**  OR model 8 = **2.7 (1.4-5.1)** | Risk |
|  | Landi (2007) | Physical activity (equal to or greater than 2 hours in the last 3 days versus less than 2 hours) versus no physical activity (reference) | OR model 1 = **0.64 (0.48-0.85)**  OR model 2 = **0.70 (0.52-0.94)** | Protective |
|  | Shah (2012) | Self-reported, hours a week (exercise, gardening, calisthenics, bicycle riding, and swimming) | HR model 2 **= 0.78 (0.68-0.90)** | Protective |
|  | Stessman (2009) | (1) less than 4 hours weekly, (2) about 4 hours weekly, (3) vigorous sports at least twice weekly (e.g., jogging or swimming) | OR model 1 = **2.12 (1.26-3.57)**  OR model 2 = **1.92 (1.11-3.33)** | Protective |
|  | Sun (2009) | Involvement in eight activities (e.g. reading newspapers, playing cards) range 0-2 (never – almost every day) | β = -.04, p < .01 | Protective |
|  |  | Taking part in a program of physical exercise or not | β = .09, p > .05 | Not associated |
| **Smoking** |  |  |  |  |
| Not smoking | Sun (2009) | Not smoking versus smoking | β = -.19, **p < .05** | Protective |
| Quitted smoking | Freedman (2008) | Quitted smoking versus never smoked | OR model 4 = 1.04 | Not associated |
| Never smoked |  | Smoking compared versus never smoked | OR model 4 = **1.24** | Risk |
| **Observed health-related measures** |  |  |  |  |
| **Cognition** |  |  |  |  |
|  | Avlund (2002b) | Digit Span, Digit Symbol, Word Fluency, Visual Reproduction, and Raven’s Progressive Matrices | OR model 1 = 1.4 (0.9-2.2)  OR model 3 = 1.2 (0.8-2.0) | Not associated |
|  | Black (2002) | Errors on a modified version of the SPMSQ (cognitive decline = two or more errors between baseline and follow-up) | OR model 2 = **1.09 (1.03-1.15)** | Risk |
|  | Corona (2013) | Mini-Mental State Exam (MMSE) | OR model 1 = 1.50 (0.88-3.30)  OR model 2 = 1.21 (0.51-2.87) | Not associated |
|  | Sun (2009) | Modified and translated MMSE | β = .02, p >.05 | Not associated |
| **Depression** |  |  |  |  |
|  | Avlund (2002b) | Center for Epidemiological Studies Depression Scale (CES-D) | OR model 1 = 1.4 (0.8-2.3)  OR model 4 = 1.2 (0.7-2.0) | Not associated |
|  | Corona (2013) | Geriatric Depression Scale (GDS) | OR model 1 = **4.05 (1.15-14.22)**  OR model 2 = 2.87 (0.64-12.71) | Unadjusted risk |
|  | Fukutomi (2013) | Depression risk, measured with 5 items (i.e. sense of fulfillment, enjoying things, and (not) feeling like a useful person) | p = .16 | Not associated |
| **Blood pressure** |  |  |  |  |
| No hypertension | Okumiya (1999) | Blood pressure measurements | OR model 1 = **0.4 (0.2-0.9)**  OR model 2 = **0.4 (0.2-0.8)** | Risk |
| Diastolic blood pressure | Sabayan (2012) | Blood pressure measurements | Model 2: p = .13 | Not associated |
| Mean arterial pressure | Sabayan (2012) | Blood pressure measurements | Model 2: **p** = **.01** | Protective |
| Pulse pressure | Sabayan (2012) | Blood pressure measurements | Model 2: **p** = **.003** | Protective |
| Systolic blood pressure | Sabayan (2012) | Blood pressure measurements | Model 2: **p** = **<.001** | Protective |
| **Frailty** |  |  |  |  |
|  | Guilley (2008) | One versus zero deficient frailty domains (sensory capacities, mobility capacities, physical pains, memory problems, and energy) | RR model 2 = 0.88 (0.24-3.25) | Not associated |
|  |  | Two versus zero deficient domains | RR model 2 = **3.56 (1.17-10.88)** | Risk |
|  |  | Three versus zero deficient domains | RR model 2 = **6.53 (2.19-19.44)** | Risk |
|  |  | > Four versus zero deficient domains | RR model 2 = **8.05 (2.69-24.08)** | Risk |
|  |  | Specific combinations of affected frailty domains | RR model 2 = 0.85 (0.24-3.26) | Not associated |
|  |  | At least two less commonly used (sensory, energy, and memory) and zero commonly used contributing factors | RR model 2 ***=* 3.95 (1.14-13.66)** | Risk |
|  |  | Other combination compared to no affected domains | RR model 2 ***=* 6.07 (2.15-17.10)** | Risk |
| **In need of long-term care** |  |  |  |  |
|  | Fukutomi (2013) | Uncertified versus specified (at high risk of requiring long-term care insurance) elderly | p = .51 | Not associated |
|  |  | Overall low score: physical strength, nutritional status, oral function, houseboundness, and cognitive function | p = .46 | Not associated |
| **Limitations in IADLs** |  |  |  |  |
|  | Corona (2013) | Limitations in IADLs versus not (using transportation, shopping, administering  one’s own money, using a telephone, controlling one’s own medications, preparing hot meals, and performing household chores) | OR model 1 **= 2.14 (1.18-3.87)**  OR model 2 = 1.67 (0.83-3.39) | Risk in unadjusted analysis only |
| **Limitation in gait/balance** |  |  |  |  |
|  | Okumiya (1999) | Timed up and go (>17 seconds) | OR model 1 **= 2.9 (1.4-6.2)** | Risk |
| **Limited muscle strength** |  |  |  |  |
| Grip strength | Corona (2013) | Dynamometer (performed twice with the dominant upper limb) | Descriptive baseline characteristics by 3-year weight change: *ns* | Not associated |
|  | Rantanen (2004) | Dynamometer fixed to the arm of an adjustable chair | OR model 2 = **2.30 (1.04-5.07)** (lowest tertile)  OR model 2 = 1.29 (0.58-2.87) (middle tertile) | Risk, lowest score |
| Elbow flexion strength |  | 90 degree angle, the hand in neutral position (thumb up), elbow supported, and wrist attached by belts | OR model 2 = **2.48 (1.06-5.79)** (lowest tertile)  OR model 2 = 1.79 (0.80-4.02)  (middle tertile) | Risk, lowest score |
| Knee extension strength |  | 60 degree angle from fully extended leg toward flexion (ankle fastened) | OR model 2 = **2.69 (1.27-5.70)** (lowest tertile)  OR model 2 = 1.06 (0.46-2.46)  (middle tertile) | Risk, lowest score |
| Trunk extension strength |  | Standing position according to a system by Viitasalo | OR model 2 = 2.16 (0.96-4.87) (lowest tertile)  OR model 2 = 2.12 (0.96-4.65) (middle tertile) | Not associated |
| Trunk flexion strength |  | Standing position according to a system by Viitasalo | OR model 2 = **3.13 (1.38-7.11)** (lowest tertile)  OR model 2 = 1.83 (0.81-4.13) (middle tertile) | Risk, lowest score |
| **Number of chronic diseases** |  |  |  |  |
|  | Avlund (2002a) | Self-reported, checked during physical examination (2-7 versus 0-1) | OR model 2 = **3.5 (1.1-11.1)** | Risk |
|  | Avlund (2002b) | Self-reported, checked during physical examination (2-7 versus 0-1) | OR model 1 = **1.8 (1.1-2.9)**  OR model 3 = 1.4 (0.8-2.4) | Mixed |
| **Other physical function limitation** |  |  |  |  |
| Functional reach | Idland (2013) | Maximum distance (cm) one can reach forward in standing position while maintaining a fixed base of support | OR model 1 ***=* 0.90 (0.84-0.97)**  OR model 2 ***=* 0.92 (0.85-0.99)**  OR model 5 = 0.94 (0.86-1.02) | Risk, but not after controlling for functional reach, climbing steps, and walking speed |
| Physical strength | Fukutomi (2013) | 5 items: go upstairs, stand up from sitting position, walk for 15 minutes, (fear of) falling | p = .43 | Not associated |
| Manual dexterity | Okumiya (1999) | Button score (>17 seconds) | OR model 1 = **2.3 (1.2-4.4)** | Risk |
| Step climbing | Idland (2013) | The ability to mount boxes of increasing heights without using a handrail | OR model 1 = **0.94 (0.90-0.97)**  OR model 3 = **0.96 (0.92-0.99)**  OR model 5 = 0.97 (0.93-1.02) | Risk, but not after controlling for functional reach, and walking speed |
| Tiredness in activities | Avlund (2002b) | Lower Limb-T Scale  (Feeling tired while performing one of four lower limb activities versus not feeling tired) | OR model 1 = **2.2 (1.1-4.2)**  OR model 2 = **2.1 (1.1-4.0)**  OR model 3 = 1.9 (0.96-3.7)  OR model 4 = **2.2 (1.1-4.2)**  OR model 5 = **2.2 (1.1-4.2)**  OR model 6 = **2.1 (1.1-4.0)**  OR model 7 = **2.2 (1.1-4.2)**  OR model 8 = **2.0 (1.0-3.9)** | Risk, but not after controlling for health factors |
|  |  | Lower Limb-T Scale  (Feeling tired while performing >2 of four lower limb activities versus not feeling tired) | OR model 1 = **2.6 (1.4-5.1)**  OR model 2 = **2.6 (1.3-5.0)**  OR model 3 = 1.9 (0.9-3.8)  OR model 4 = **2.6 (1.3-5.0)**  OR model 5 = **2.6 (1.3-5.0)**  OR model 6 = **2.4 (1.2-4.7)**  OR model 7 = **2.5 (1.3-5.0)**  OR model 8 = **2.1 (1.0-4.2)** | Risk, but not after controlling for health factors |
| Walking speed | Idland (2013) | Distance of 29 meters where the participant walked a corridor at comfortable speed | OR model 1 = **0.01 (0.01-0.17)**  OR model 4 = **0.40 (0.02-0.69)**  OR model 5 = **0.40 (0.02-0.69)** | Risk |
| **Weight** |  |  |  |  |
| Weight gain | Corona (2013) | ≥ 5% weight gain, **using a calibrated scale** | OR model 1 = **2.42 (1.14-5.15)**  OR model 2 = **2.42 (1.11-5.29)** | Risk |
| Weight loss | Corona (2013) | ≥5% weight loss, **using a calibrated scale** | OR model 1 = **3.02 (1.22-7.48)**  OR model 2 = 1.66 (0.52-5.36) | Unadjusted risk |
| **Vitamin status** |  |  |  |  |
|  | Houston (2011) | 25(OH)D ng/ML <20.0 | HR model 2 = 1.18 (0.75 – 1.84)  HR model 3 = 0.98 (0.61 – 1.56) | Not associated |
|  |  | 25(OH)D ng/ML 20.0-29.9 | HR model 2 = 0.79 (0.51-1.22)  HR model 3 = 0.75 (0.48-1.18) | Not associated |
| **Self-reported health-related measure** |  |  |  |  |
| **Falls** |  |  |  |  |
|  | Donald (1999) | Fallers versus non-fallers | p = .09 for first wave  p = .64 for second wave | Not associated |
| **Cognition** |  |  |  |  |
|  | Fukutomi (2013) | 3 items (e.g. “do others point you to forgetfulness”) | p = .47 | Not associated |
| **Hearing** |  |  |  |  |
|  | Freedman (2008) | Good versus excellent/very good hearing (self-rated) | OR model 4 = 1.08 | Not associated |
|  |  | Fair/poor versus excellent/very good hearing (self-rated) | OR model 4 = **1.44** | Risk |
| **Medication** |  |  |  |  |
|  | Idland (2013) | Number of medications, self-reported | p = .32, not examined in logistic regression models | Not associated |
| **Pain** |  |  |  |  |
|  | Landi (2009) | Daily versus no daily pain | OR model 1 = **1.99 (1.01-4.28)**  OR model 2 = 1.87 (0.92-4.26) | Risk in unadjusted analysis only |
|  |  | Single site versus no daily pain | OR model 1 = 1.04 (0.27-4.02)  OR model 2 = 1.33 (0.43-5.37) | Not associated |
|  |  | Multiple sites versus no daily pain | OR model 1 = **2.34 (1.11-4.94)**  OR model 2 = 2.10 (0.99-4.97) | Risk in unadjusted analysis only |
|  |  | Mild versus no daily pain | OR model 1 = 1.31 (0.58-2.95)  OR model 2 = 1.35 (0.65-3.28) | Not associated |
|  |  | Moderate to severe versus no daily pain | OR model 1 = **5.69 (2.10-15.39)**  OR model 2 = **6.94 (2.00-23.01)** | Risk |
| **Peak stature** |  |  |  |  |
|  | Freedman (2008) | Below average or not | OR model 2 = **1.55**  OR model 3 = 1.49  OR model 4 = 1.40 | Mixed |
| **Self-rated health** |  |  |  |  |
|  | Avlund (2002b) | Very good/good versus fair/poor | OR model 1 = **2.1 (1.3-3.4)**  OR model 3 = 1.6 (0.9-2.6)  OR model 8 = 1.6 (0.96-2.7) | Risk in unadjusted analysis only |
|  | Idland (2013) | Self-reported, 1-5 (best to worst) | OR model 1 = **1.85 (1.12-3.03)**  OR model 2 = 1.52 (0.91-2.55)  OR model 3 = 1.48 (0.91-2.55)  OR model 4 = 1.50 (0.89-1.12) | Risk in unadjusted analysis only |
|  | Sun (2009) | One question, 1-5 (very bad – very good) | β = -.03, p > .05 | Not associated |
|  | Idland (2013) | General health 0-60 (no distress – severe distress) | p = 0.85, not examined in logistic regression models | Not associated |
| **Self-rated health as a child** |  |  |  |  |
|  | Freedman (2008) | Fair/poor versus excellent self-rated health as a child | OR model 2 = **1.80**  OR model 3 = **1.71**  OR model 4 = 1.40 | Risk, but no longer after controlling for late-life factors |
|  |  | Good versus excellent self-rated health as a child | OR model 2 = **1.42**  OR model 3 = **1.36**  OR model 4 = **1.20** | Risk |
|  |  | Very good versus excellent self-rated health as a child | OR model 2 = **1.10**  OR model 3 = 1.08  OR model 4 = 0.98 | Risk, but no longer after controlling for mid- and late-life factors |
| **Subjective well-being** |  |  |  |  |
|  | Sun (2009) | Six-item scale that assessed quality of life, attitude toward life, agitation, loneliness, attitude toward aging, and happiness. | β = .01, p > .05 | Not associated |
| **Vision** |  |  |  |  |
|  | Freedman (2008) | Good vision compared to excellent/very good | OR model 4 = **1.12** | Risk |
|  |  | Fair/poor vision compared to excellent/very good | OR model 4 = **2.35** | Risk |
| **Weight / nutrition** |  |  |  |  |
| Obesity | Freedman (2008) | Obesity (BMI), based on self-reported height and weight | OR model 4 = **1.56** | Risk |
| Weight loss | Corona (2013) | Unintentional weight loss: report of losing more than 3 kilograms in the last 12 months without dieting. | OR model 1 = 1.92 (0.80-4.62)  OR model 2 = 1.77 (0.59-5.31) | Not associated |
|  | Fukutomi (2013) | Decline in weight (BMI) | “Not further examined” | Not associated |
|  | Landi (2010) | Anorexia of ageing (= presence of decreased food intake or the presence of poor appetite) | Model 1: **p = .02**  Model 2: p = .26 | Unadjusted risk |
| **Other** |  |  |  |  |
| **Proxy response** |  |  |  |  |
|  | Freedman (2008) | Interview provided by proxy versus self-report | OR model 1 = **4.95**  OR model 2 = **4.67**  OR model 3 = **4.36**  OR model 4 = **4.18** | Risk |

* Model 1 = unadjusted analyses for all studies. For the specification of other models per study, see below:

| **First author (year)** | **Adjusted models** |
| --- | --- |
| Avlund (2002a) | 1. Chronic diseases, home help, and locality |
| Avlund (2002b) | 1. Sex and locality 2. Health factors 3. Depressive symptoms 4. Housing tenure 5. Social relations 6. Physical activity 7. Physical activity or self-rated health (final model) |
| Avlund (2004) | 1. Social relations variables that were related to the specific outcome measures in the unadjusted analyses 2. Social relations variables that were related to the specific outcome measures in model 2 3. Model 3 + live alone 4. Variables model 4 if related to outcome, and chronic diseases, cognitive function, depressive symptoms, and locality |
| Black (2002) | 1. Sociodemographics, chronic health conditions, cognitive status and functional decline |
| Corona (2013) | 1. Factors significant in unadjusted analyses (age, IADL, cognition, depression, unintentional weight loss) |
| Donald (1999) | 1. Unclear |
| Freedman (2008) | 1. Early-life factors (race/ethnicity, region of birth, mother's education, childhood SES, childhood self-rated health and low peak stature) 2. Plus mid-life factors (education, veteran, lifetime occupation) 3. Plus late-life factors (marital status, region of residence, income, assets, smokers, chronic conditions, obesity, vision and hearing) |
| Fukutomi (2013) | - |
| Gu (2011) | 1. Socio-demographic variables 2. Socio-demographic variables and “all other controlling variables” (family and social support/connections, alcohol consumption, smoking, diet, exercise, cognition, self-reported health, depression, chronic disease, hearing and visual impairment, lower and upper extremities, survivors verses decedents) |
| Guilley (2008) | 1. Controlled for age, gender, socioeconomic status, and geographical area |
| Houston (2011) | 1. Age, sex, race, education, field center, season 2. Model 1 + BMI, walking for physical activity, smoking, alcohol use, creatinine, depression, cognition, diabetes mellitus, osteoporosis, cardiovascular disease |
| Idland (2013) | 1. Functional reach and covariates 2. Climbing steps and covariates 3. Walking speed and covariates 4. Functional reach, climbing steps, walking speed and covariates |
| Jiang (2002) | - |
| Landi (2007) | 1. Age, gender, cognitive performance, vision, heart disease, heart failure, stroke, vascular disease, COPD, osteoarthritis, depression, number of medications, country |
| Landi (2009) | 1. Age, gender, education, BMI, osteoarthritis, hypertension, depression, and number of diseases |
| Landi (2010) | 1. Age, gender, BMI, number of diseases, depression, heart failure, lung diseases |
| Li (2009) | 1. Sociodemographic characteristics, availability of children, health status of the respondent |
| Moody-Ayers (2005) | 1. Sex and age 2. Sex, age, hypertension, diabetes, cancer, lung and heart disease, and stroke 3. Sex, age, and smoking status 4. Sex, age, and self-rated health 5. Sex, age, and socioeconomic status: 6. Sex, age, and cognitive function |
| Okumiya (1999) | 1. Up and go, Button score (manual dexterity), age, hypertension |
| Rantanen (2002) | 1. Body height, body weight, research site, gender |
| Sabayan (2012) | 1. Sex, education, smoking status, alcohol intake, history of stroke, types of antihypertensive medications,, diabetes mellitus, history of cardiovascular disease, baseline physical and cognitive scores |
| Shah (2012) | 1. Age, sex, and education |
| Stessman (2009) | 1. Sex, marital status, educational status, self-rated health, physical activity level, chronic pain, loneliness |
| Stessman (2014) | 1. Sex, financial status, origin, BMI, smoking pack-years, ease of performance in ADLs, hypertension, ischemic heart disease, diabetes mellitus, history of neoplasm, renal disease |
| Sun (2009) | 1. Baseline covariates |
